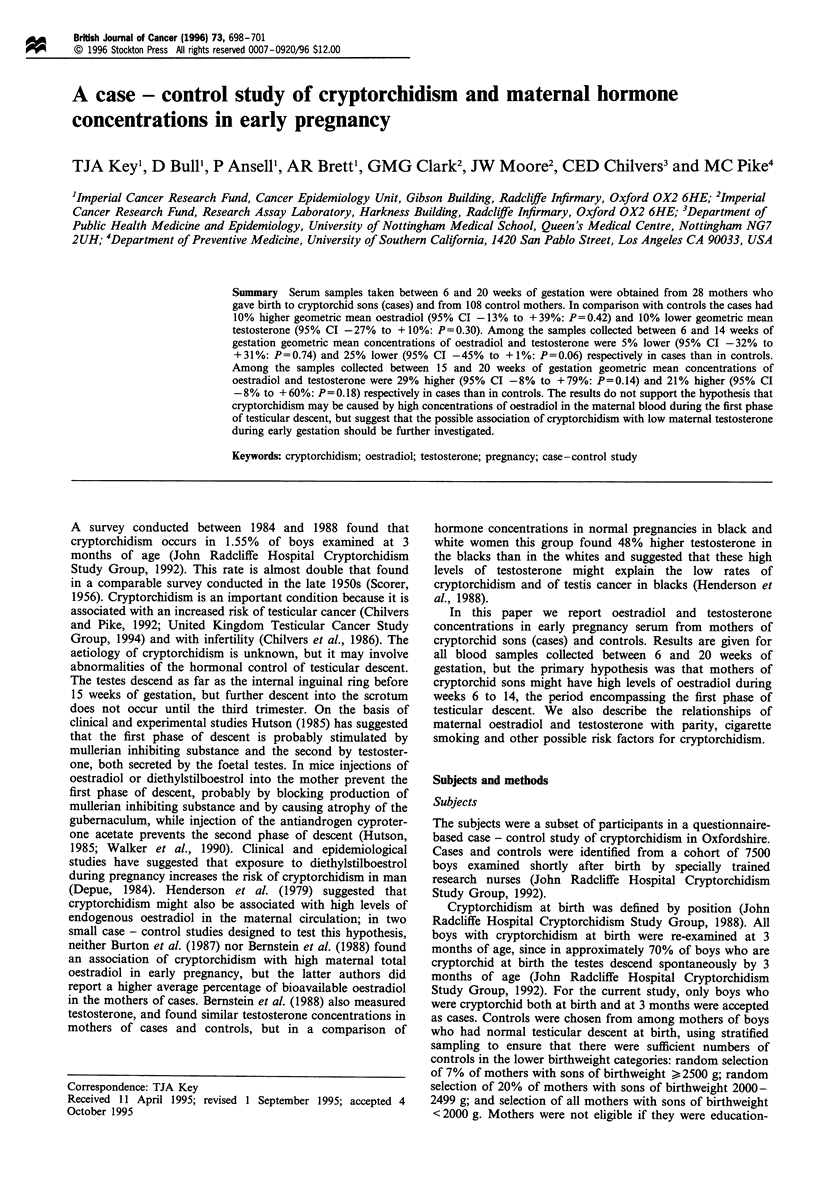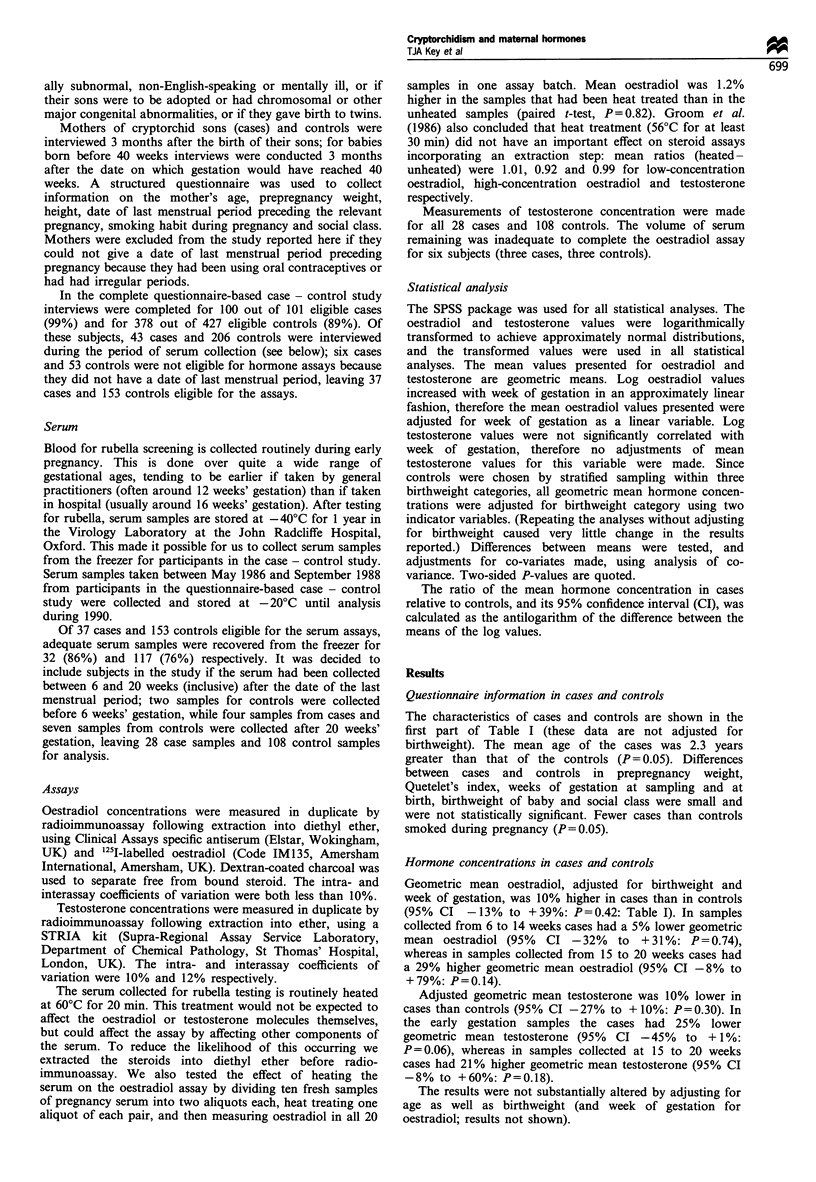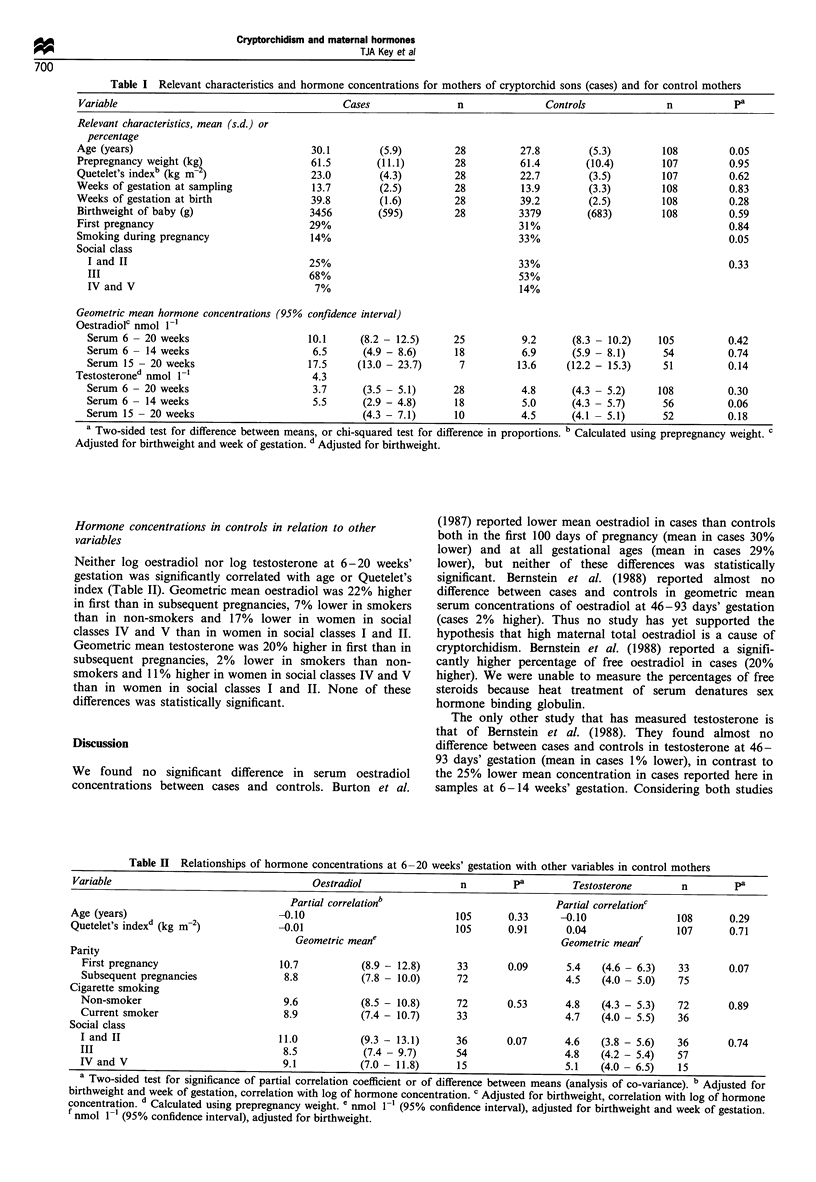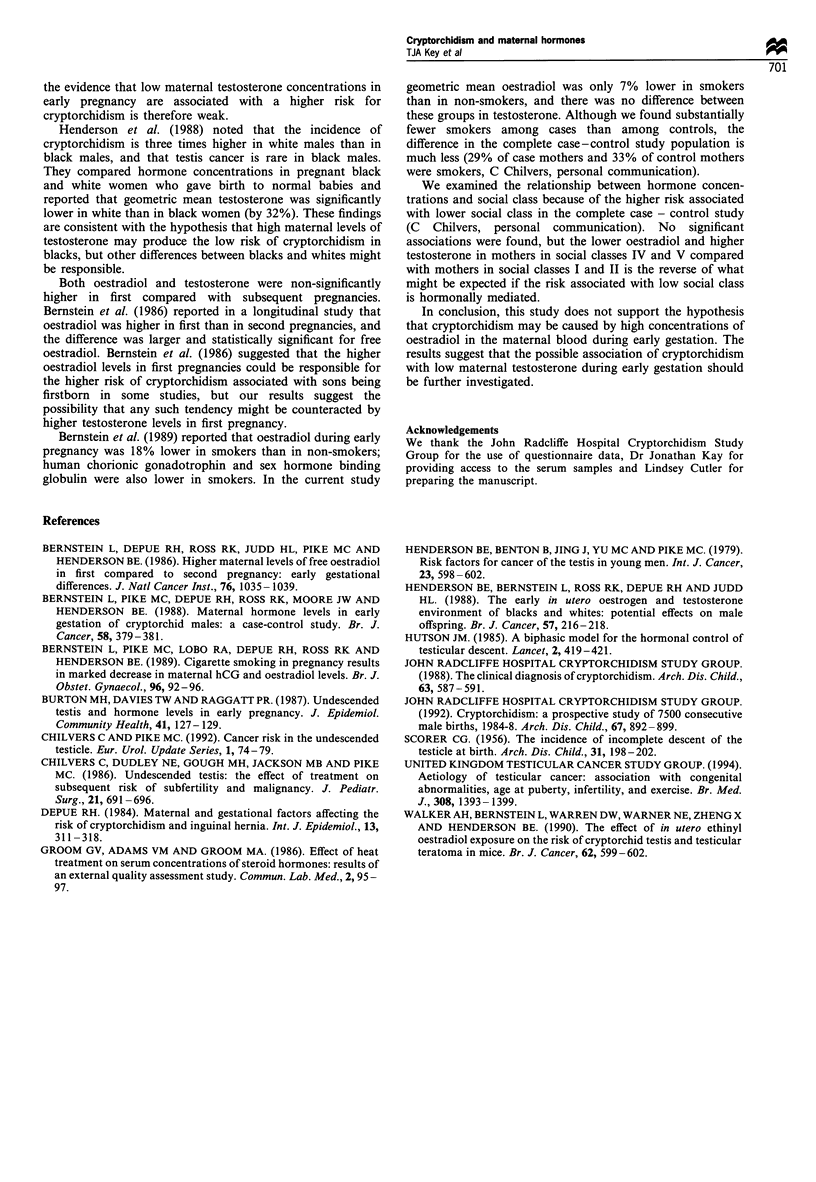# A case-control study of cryptorchidism and maternal hormone concentrations in early pregnancy.

**DOI:** 10.1038/bjc.1996.121

**Published:** 1996-03

**Authors:** T. J. Key, D. Bull, P. Ansell, A. R. Brett, G. M. Clark, J. W. Moore, C. E. Chilvers, M. C. Pike

**Affiliations:** Imperial Cancer Research Fund, Cancer Epidemiology Unit, Radcliffe Infirmary, Oxford, UK.

## Abstract

Serum samples taken between 6 and 20 weeks of gestation were obtained from 28 mothers who gave birth to cryptorchid sons (cases) and from 108 control mothers. In comparison with controls the cases had 10% higher geometric mean oestradiol (95% CI -13% to +39%: P=0.42) and 10% lower geometric mean testosterone (95% CI -27% to +10%: P=0.30). Among the samples collected between 6 and 14 weeks of gestation geometric mean concentrations of oestradiol and testosterone were 5% lower (95% CI -32% to +31%: P=0.74) and 25% lower (95% CI -45% to +1%: P=0.06) respectively in cases than in controls. Among the samples collected between 15 and 20 weeks of gestation geometric mean concentrations of oestradiol and testosterone were 29% higher (95% CI -8% to +79%: P=0.14) and 21% higher (95% CI -8% to +60%: P=0.18) respectively in cases than in controls. The results do not support the hypothesis that cryptorchidism may be caused by high concentrations of oestradiol in the maternal blood during the first phase of testicular descent, but suggest that the possible association of cryptorchidism with low maternal testosterone during early gestation should be further investigated.


British Journal of Cancer (1996) 73, 698-701
© 1996 Stockton Press All rights reserved 0007-0920/96 $12.00

### A case - control study of cryptorchidism and maternal hormone concentrations in early pregnancy

TJA Key[1], D Bull[1], P Ansell[1], AR Brett[1], GMG Clark[2], JW Moore[2], CED Chilvers[3] and MC Pike[4]

[1]*Imperial Cancer Research Fund, Cancer Epidemiology Unit, Gibson Building, Radcliffe Infirmary, Oxford OX2 6HE;* [2]*Imperial Cancer Research Fund, Research Assay Laboratory, Harkness Building, Radcliffe Infirmary, Oxford OX2 6HE;* [3]*Department of Public Health Medicine and Epidemiology, University of Nottingham Medical School, Queen's Medical Centre, Nottingham NG7 2UH;* [4]*Department of Preventive Medicine, University of Southern California, 1420 San Pablo Street, Los Angeles CA 90033, USA*

**Summary** Serum samples taken between 6 and 20 weeks of gestation were obtained from 28 mothers who gave birth to cryptorchid sons (cases) and from 108 control mothers. In comparison with controls the cases had 10% higher geometric mean oestradiol (95% CI −13% to +39%: $P=0.42$) and 10% lower geometric mean testosterone (95% CI −27% to +10%: $P=0.30$). Among the samples collected between 6 and 14 weeks of gestation geometric mean concentrations of oestradiol and testosterone were 5% lower (95% CI −32% to +31%: $P=0.74$) and 25% lower (95% CI −45% to +1%: $P=0.06$) respectively in cases than in controls. Among the samples collected between 15 and 20 weeks of gestation geometric mean concentrations of oestradiol and testosterone were 29% higher (95% CI −8% to +79%: $P=0.14$) and 21% higher (95% CI −8% to +60%: $P=0.18$) respectively in cases than in controls. The results do not support the hypothesis that cryptorchidism may be caused by high concentrations of oestradiol in the maternal blood during the first phase of testicular descent, but suggest that the possible association of cryptorchidism with low maternal testosterone during early gestation should be further investigated.

**Keywords:** cryptorchidism; oestradiol; testosterone; pregnancy; case-control study

A survey conducted between 1984 and 1988 found that cryptorchidism occurs in 1.55% of boys examined at 3 months of age (John Radcliffe Hospital Cryptorchidism Study Group, 1992). This rate is almost double that found in a comparable survey conducted in the late 1950s (Scorer, 1956). Cryptorchidism is an important condition because it is associated with an increased risk of testicular cancer (Chilvers and Pike, 1992; United Kingdom Testicular Cancer Study Group, 1994) and with infertility (Chilvers *et al.*, 1986). The aetiology of cryptorchidism is unknown, but it may involve abnormalities of the hormonal control of testicular descent. The testes descend as far as the internal inguinal ring before 15 weeks of gestation, but further descent into the scrotum does not occur until the third trimester. On the basis of clinical and experimental studies Hutson (1985) has suggested that the first phase of descent is probably stimulated by mullerian inhibiting substance and the second by testosterone, both secreted by the foetal testes. In mice injections of oestradiol or diethylstilboestrol into the mother prevent the first phase of descent, probably by blocking production of mullerian inhibiting substance and by causing atrophy of the gubernaculum, while injection of the antiandrogen cyproterone acetate prevents the second phase of descent (Hutson, 1985; Walker *et al.*, 1990). Clinical and epidemiological studies have suggested that exposure to diethylstilboestrol during pregnancy increases the risk of cryptorchidism in man (Depue, 1984). Henderson *et al.* (1979) suggested that cryptorchidism might also be associated with high levels of endogenous oestradiol in the maternal circulation; in two small case - control studies designed to test this hypothesis, neither Burton *et al.* (1987) nor Bernstein *et al.* (1988) found an association of cryptorchidism with high maternal total oestradiol in early pregnancy, but the latter authors did report a higher average percentage of bioavailable oestradiol in the mothers of cases. Bernstein *et al.* (1988) also measured testosterone, and found similar testosterone concentrations in mothers of cases and controls, but in a comparison of hormone concentrations in normal pregnancies in black and white women this group found 48% higher testosterone in the blacks than in the whites and suggested that these high levels of testosterone might explain the low rates of cryptorchidism and of testis cancer in blacks (Henderson *et al.*, 1988).

In this paper we report oestradiol and testosterone concentrations in early pregnancy serum from mothers of cryptorchid sons (cases) and controls. Results are given for all blood samples collected between 6 and 20 weeks of gestation, but the primary hypothesis was that mothers of cryptorchid sons might have high levels of oestradiol during weeks 6 to 14, the period encompassing the first phase of testicular descent. We also describe the relationships of maternal oestradiol and testosterone with parity, cigarette smoking and other possible risk factors for cryptorchidism.

### Subjects and methods

### *Subjects*

The subjects were a subset of participants in a questionnaire-based case - control study of cryptorchidism in Oxfordshire. Cases and controls were identified from a cohort of 7500 boys examined shortly after birth by specially trained research nurses (John Radcliffe Hospital Cryptorchidism Study Group, 1992).

Cryptorchidism at birth was defined by position (John Radcliffe Hospital Cryptorchidism Study Group, 1988). All boys with cryptorchidism at birth were re-examined at 3 months of age, since in approximately 70% of boys who are cryptorchid at birth the testes descend spontaneously by 3 months of age (John Radcliffe Hospital Cryptorchidism Study Group, 1992). For the current study, only boys who were cryptorchid both at birth and at 3 months were accepted as cases. Controls were chosen from among mothers of boys who had normal testicular descent at birth, using stratified sampling to ensure that there were sufficient numbers of controls in the lower birthweight categories: random selection of 7% of mothers with sons of birthweight ⩾2500 g; random selection of 20% of mothers with sons of birthweight 2000-2499 g; and selection of all mothers with sons of birthweight <2000 g. Mothers were not eligible if they were education-

Correspondence: TJA Key
Received 11 April 1995; revised 1 September 1995; accepted 4 October 1995

ally subnormal, non-English-speaking or mentally ill, or if their sons were to be adopted or had chromosomal or other major congenital abnormalities, or if they gave birth to twins.

Mothers of cryptorchid sons (cases) and controls were interviewed 3 months after the birth of their sons; for babies born before 40 weeks interviews were conducted 3 months after the date on which gestation would have reached 40 weeks. A structured questionnaire was used to collect information on the mother's age, prepregnancy weight, height, date of last menstrual period preceding the relevant pregnancy, smoking habit during pregnancy and social class. Mothers were excluded from the study reported here if they could not give a date of last menstrual period preceding pregnancy because they had been using oral contraceptives or had had irregular periods.

In the complete questionnaire-based case – control study interviews were completed for 100 out of 101 eligible cases (99%) and for 378 out of 427 eligible controls (89%). Of these subjects, 43 cases and 206 controls were interviewed during the period of serum collection (see below); six cases and 53 controls were not eligible for hormone assays because they did not have a date of last menstrual period, leaving 37 cases and 153 controls eligible for the assays.

### *Serum*

Blood for rubella screening is collected routinely during early pregnancy. This is done over quite a wide range of gestational ages, tending to be earlier if taken by general practitioners (often around 12 weeks' gestation) than if taken in hospital (usually around 16 weeks' gestation). After testing for rubella, serum samples are stored at −40°C for 1 year in the Virology Laboratory at the John Radcliffe Hospital, Oxford. This made it possible for us to collect serum samples from the freezer for participants in the case – control study. Serum samples taken between May 1986 and September 1988 from participants in the questionnaire-based case – control study were collected and stored at −20°C until analysis during 1990.

Of 37 cases and 153 controls eligible for the serum assays, adequate serum samples were recovered from the freezer for 32 (86%) and 117 (76%) respectively. It was decided to include subjects in the study if the serum had been collected between 6 and 20 weeks (inclusive) after the date of the last menstrual period; two samples for controls were collected before 6 weeks' gestation, while four samples from cases and seven samples from controls were collected after 20 weeks' gestation, leaving 28 case samples and 108 control samples for analysis.

### *Assays*

Oestradiol concentrations were measured in duplicate by radioimmunoassay following extraction into diethyl ether, using Clinical Assays specific antiserum (Elstar, Wokingham, UK) and $^{125}$I-labelled oestradiol (Code IM135, Amersham International, Amersham, UK). Dextran-coated charcoal was used to separate free from bound steroid. The intra- and interassay coefficients of variation were both less than 10%.

Testosterone concentrations were measured in duplicate by radioimmunoassay following extraction into ether, using a STRIA kit (Supra-Regional Assay Service Laboratory, Department of Chemical Pathology, St Thomas' Hospital, London, UK). The intra- and interassay coefficients of variation were 10% and 12% respectively.

The serum collected for rubella testing is routinely heated at 60°C for 20 min. This treatment would not be expected to affect the oestradiol or testosterone molecules themselves, but could affect the assay by affecting other components of the serum. To reduce the likelihood of this occurring we extracted the steroids into diethyl ether before radioimmunoassay. We also tested the effect of heating the serum on the oestradiol assay by dividing ten fresh samples of pregnancy serum into two aliquots each, heat treating one aliquot of each pair, and then measuring oestradiol in all 20 samples in one assay batch. Mean oestradiol was 1.2% higher in the samples that had been heat treated than in the unheated samples (paired $t$-test, $P=0.82$). Groom *et al.* (1986) also concluded that heat treatment (56°C for at least 30 min) did not have an important effect on steroid assays incorporating an extraction step: mean ratios (heated−unheated) were 1.01, 0.92 and 0.99 for low-concentration oestradiol, high-concentration oestradiol and testosterone respectively.

Measurements of testosterone concentration were made for all 28 cases and 108 controls. The volume of serum remaining was inadequate to complete the oestradiol assay for six subjects (three cases, three controls).

### *Statistical analysis*

The SPSS package was used for all statistical analyses. The oestradiol and testosterone values were logarithmically transformed to achieve approximately normal distributions, and the transformed values were used in all statistical analyses. The mean values presented for oestradiol and testosterone are geometric means. Log oestradiol values increased with week of gestation in an approximately linear fashion, therefore the mean oestradiol values presented were adjusted for week of gestation as a linear variable. Log testosterone values were not significantly correlated with week of gestation, therefore no adjustments of mean testosterone values for this variable were made. Since controls were chosen by stratified sampling within three birthweight categories, all geometric mean hormone concentrations were adjusted for birthweight category using two indicator variables. (Repeating the analyses without adjusting for birthweight caused very little change in the results reported.) Differences between means were tested, and adjustments for co-variates made, using analysis of co-variance. Two-sided $P$-values are quoted.

The ratio of the mean hormone concentration in cases relative to controls, and its 95% confidence interval (CI), was calculated as the antilogarithm of the difference between the means of the log values.

### Results

### *Questionnaire information in cases and controls*

The characteristics of cases and controls are shown in the first part of Table I (these data are not adjusted for birthweight). The mean age of the cases was 2.3 years greater than that of the controls ($P=0.05$). Differences between cases and controls in prepregnancy weight, Quetelet's index, weeks of gestation at sampling and at birth, birthweight of baby and social class were small and were not statistically significant. Fewer cases than controls smoked during pregnancy ($P=0.05$).

### *Hormone concentrations in cases and controls*

Geometric mean oestradiol, adjusted for birthweight and week of gestation, was 10% higher in cases than in controls (95% CI −13% to +39%: $P=0.42$: Table I). In samples collected from 6 to 14 weeks cases had a 5% lower geometric mean oestradiol (95% CI −32% to +31%: $P=0.74$), whereas in samples collected from 15 to 20 weeks cases had a 29% higher geometric mean oestradiol (95% CI −8% to +79%: $P=0.14$).

Adjusted geometric mean testosterone was 10% lower in cases than controls (95% CI −27% to +10%: $P=0.30$). In the early gestation samples the cases had 25% lower geometric mean testosterone (95% CI −45% to +1%: $P=0.06$), whereas in samples collected at 15 to 20 weeks cases had 21% higher geometric mean testosterone (95% CI −8% to +60%: $P=0.18$).

The results were not substantially altered by adjusting for age as well as birthweight (and week of gestation for oestradiol; results not shown).

**Table I** Relevant characteristics and hormone concentrations for mothers of cryptorchid sons (cases) and for control mothers

| *Variable* | *Cases* | | n | *Controls* | | n | P[a] |
|---|---|---|---|---|---|---|---|
| *Relevant characteristics, mean (s.d.) or percentage* | | | | | | | |
| Age (years) | 30.1 | (5.9) | 28 | 27.8 | (5.3) | 108 | 0.05 |
| Prepregnancy weight (kg) | 61.5 | (11.1) | 28 | 61.4 | (10.4) | 107 | 0.95 |
| Quetelet's index[b] (kg $m^{-2}$) | 23.0 | (4.3) | 28 | 22.7 | (3.5) | 107 | 0.62 |
| Weeks of gestation at sampling | 13.7 | (2.5) | 28 | 13.9 | (3.3) | 108 | 0.83 |
| Weeks of gestation at birth | 39.8 | (1.6) | 28 | 39.2 | (2.5) | 108 | 0.28 |
| Birthweight of baby (g) | 3456 | (595) | 28 | 3379 | (683) | 108 | 0.59 |
| First pregnancy | 29% | | | 31% | | | 0.84 |
| Smoking during pregnancy | 14% | | | 33% | | | 0.05 |
| Social class | | | | | | | |
| I and II | 25% | | | 33% | | | 0.33 |
| III | 68% | | | 53% | | | |
| IV and V | 7% | | | 14% | | | |
| *Geometric mean hormone concentrations (95% confidence interval)* | | | | | | | |
| Oestradiol[c] nmol $l^{-1}$ | | | | | | | |
| Serum 6 – 20 weeks | 10.1 | (8.2 – 12.5) | 25 | 9.2 | (8.3 – 10.2) | 105 | 0.42 |
| Serum 6 – 14 weeks | 6.5 | (4.9 – 8.6) | 18 | 6.9 | (5.9 – 8.1) | 54 | 0.74 |
| Serum 15 – 20 weeks | 17.5 | (13.0 – 23.7) | 7 | 13.6 | (12.2 – 15.3) | 51 | 0.14 |
| Testosterone[d] nmol $l^{-1}$ | 4.3 | | | | | | |
| Serum 6 – 20 weeks | 3.7 | (3.5 – 5.1) | 28 | 4.8 | (4.3 – 5.2) | 108 | 0.30 |
| Serum 6 – 14 weeks | 5.5 | (2.9 – 4.8) | 18 | 5.0 | (4.3 – 5.7) | 56 | 0.06 |
| Serum 15 – 20 weeks | | (4.3 – 7.1) | 10 | 4.5 | (4.1 – 5.1) | 52 | 0.18 |

[a] Two-sided test for difference between means, or chi-squared test for difference in proportions. [b] Calculated using prepregnancy weight. [c] Adjusted for birthweight and week of gestation. [d] Adjusted for birthweight.

### *Hormone concentrations in controls in relation to other variables*

Neither log oestradiol nor log testosterone at 6–20 weeks' gestation was significantly correlated with age or Quetelet's index (Table II). Geometric mean oestradiol was 22% higher in first than in subsequent pregnancies, 7% lower in smokers than in non-smokers and 17% lower in women in social classes IV and V than in women in social classes I and II. Geometric mean testosterone was 20% higher in first than in subsequent pregnancies, 2% lower in smokers than non-smokers and 11% higher in women in social classes IV and V than in women in social classes I and II. None of these differences was statistically significant.

### Discussion

We found no significant difference in serum oestradiol concentrations between cases and controls. Burton *et al.* (1987) reported lower mean oestradiol in cases than controls both in the first 100 days of pregnancy (mean in cases 30% lower) and at all gestational ages (mean in cases 29% lower), but neither of these differences was statistically significant. Bernstein *et al.* (1988) reported almost no difference between cases and controls in geometric mean serum concentrations of oestradiol at 46–93 days' gestation (cases 2% higher). Thus no study has yet supported the hypothesis that high maternal total oestradiol is a cause of cryptorchidism. Bernstein *et al.* (1988) reported a significantly higher percentage of free oestradiol in cases (20% higher). We were unable to measure the percentages of free steroids because heat treatment of serum denatures sex hormone binding globulin.

The only other study that has measured testosterone is that of Bernstein *et al.* (1988). They found almost no difference between cases and controls in testosterone at 46–93 days' gestation (mean in cases 1% lower), in contrast to the 25% lower mean concentration in cases reported here in samples at 6–14 weeks' gestation. Considering both studies

**Table II** Relationships of hormone concentrations at 6–20 weeks' gestation with other variables in control mothers

| *Variable* | *Oestradiol* | | n | P[a] | *Testosterone* | | n | P[a] |
|---|---|---|---|---|---|---|---|---|
| | *Partial correlation[b]* | | | | *Partial correlation[c]* | | | |
| Age (years) | –0.10 | | 105 | 0.33 | –0.10 | | 108 | 0.29 |
| Quetelet's index[d] (kg $m^{-2}$) | –0.01 | | 105 | 0.91 | 0.04 | | 107 | 0.71 |
| | *Geometric mean[e]* | | | | *Geometric mean[f]* | | | |
| Parity | | | | | | | | |
| First pregnancy | 10.7 | (8.9 – 12.8) | 33 | 0.09 | 5.4 | (4.6 – 6.3) | 33 | 0.07 |
| Subsequent pregnancies | 8.8 | (7.8 – 10.0) | 72 | | 4.5 | (4.0 – 5.0) | 75 | |
| Cigarette smoking | | | | | | | | |
| Non-smoker | 9.6 | (8.5 – 10.8) | 72 | 0.53 | 4.8 | (4.3 – 5.3) | 72 | 0.89 |
| Current smoker | 8.9 | (7.4 – 10.7) | 33 | | 4.7 | (4.0 – 5.5) | 36 | |
| Social class | | | | | | | | |
| I and II | 11.0 | (9.3 – 13.1) | 36 | 0.07 | 4.6 | (3.8 – 5.6) | 36 | 0.74 |
| III | 8.5 | (7.4 – 9.7) | 54 | | 4.8 | (4.2 – 5.4) | 57 | |
| IV and V | 9.1 | (7.0 – 11.8) | 15 | | 5.1 | (4.0 – 6.5) | 15 | |

[a] Two-sided test for significance of partial correlation coefficient or of difference between means (analysis of co-variance). [b] Adjusted for birthweight and week of gestation, correlation with log of hormone concentration. [c] Adjusted for birthweight, correlation with log of hormone concentration. [d] Calculated using prepregnancy weight. [e] nmol $l^{-1}$ (95% confidence interval), adjusted for birthweight and week of gestation. [f] nmol $l^{-1}$ (95% confidence interval), adjusted for birthweight.

the evidence that low maternal testosterone concentrations in early pregnancy are associated with a higher risk for cryptorchidism is therefore weak.

Henderson *et al.* (1988) noted that the incidence of cryptorchidism is three times higher in white males than in black males, and that testis cancer is rare in black males. They compared hormone concentrations in pregnant black and white women who gave birth to normal babies and reported that geometric mean testosterone was significantly lower in white than in black women (by 32%). These findings are consistent with the hypothesis that high maternal levels of testosterone may produce the low risk of cryptorchidism in blacks, but other differences between blacks and whites might be responsible.

Both oestradiol and testosterone were non-significantly higher in first compared with subsequent pregnancies. Bernstein *et al.* (1986) reported in a longitudinal study that oestradiol was higher in first than in second pregnancies, and the difference was larger and statistically significant for free oestradiol. Bernstein *et al.* (1986) suggested that the higher oestradiol levels in first pregnancies could be responsible for the higher risk of cryptorchidism associated with sons being firstborn in some studies, but our results suggest the possibility that any such tendency might be counteracted by higher testosterone levels in first pregnancy.

Bernstein *et al.* (1989) reported that oestradiol during early pregnancy was 18% lower in smokers than in non-smokers; human chorionic gonadotrophin and sex hormone binding globulin were also lower in smokers. In the current study geometric mean oestradiol was only 7% lower in smokers than in non-smokers, and there was no difference between these groups in testosterone. Although we found substantially fewer smokers among cases than among controls, the difference in the complete case–control study population is much less (29% of case mothers and 33% of control mothers were smokers, C Chilvers, personal communication).

We examined the relationship between hormone concentrations and social class because of the higher risk associated with lower social class in the complete case – control study (C Chilvers, personal communication). No significant associations were found, but the lower oestradiol and higher testosterone in mothers in social classes IV and V compared with mothers in social classes I and II is the reverse of what might be expected if the risk associated with low social class is hormonally mediated.

In conclusion, this study does not support the hypothesis that cryptorchidism may be caused by high concentrations of oestradiol in the maternal blood during early gestation. The results suggest that the possible association of cryptorchidism with low maternal testosterone during early gestation should be further investigated.

**Acknowledgements**
We thank the John Radcliffe Hospital Cryptorchidism Study Group for the use of questionnaire data, Dr Jonathan Kay for providing access to the serum samples and Lindsey Cutler for preparing the manuscript.

### References

BERNSTEIN L, DEPUE RH, ROSS RK, JUDD HL, PIKE MC AND HENDERSON BE. (1986). Higher maternal levels of free oestradiol in first compared to second pregnancy: early gestational differences. *J. Natl Cancer Inst.*, **76,** 1035–1039.

BERNSTEIN L, PIKE MC, DEPUE RH, ROSS RK, MOORE JW AND HENDERSON BE. (1988). Maternal hormone levels in early gestation of cryptorchid males: a case-control study. *Br. J. Cancer*, **58,** 379–381.

BERNSTEIN L, PIKE MC, LOBO RA, DEPUE RH, ROSS RK AND HENDERSON BE. (1989). Cigarette smoking in pregnancy results in marked decrease in maternal hCG and oestradiol levels. *Br. J. Obstet. Gynaecol.*, **96,** 92–96.

BURTON MH, DAVIES TW AND RAGGATT PR. (1987). Undescended testis and hormone levels in early pregnancy. *J. Epidemiol. Community Health*, **41,** 127–129.

CHILVERS C AND PIKE MC. (1992). Cancer risk in the undescended testicle. *Eur. Urol. Update Series*, **1,** 74–79.

CHILVERS C, DUDLEY NE, GOUGH MH, JACKSON MB AND PIKE MC. (1986). Undescended testis: the effect of treatment on subsequent risk of subfertility and malignancy. *J. Pediatr. Surg.*, **21,** 691–696.

DEPUE RH. (1984). Maternal and gestational factors affecting the risk of cryptorchidism and inguinal hernia. *Int. J. Epidemiol.*, **13,** 311–318.

GROOM GV, ADAMS VM AND GROOM MA. (1986). Effect of heat treatment on serum concentrations of steroid hormones: results of an external quality assessment study. *Commun. Lab. Med.*, **2,** 95–97.

HENDERSON BE, BENTON B, JING J, YU MC AND PIKE MC. (1979). Risk factors for cancer of the testis in young men. *Int. J. Cancer*, **23,** 598–602.

HENDERSON BE, BERNSTEIN L, ROSS RK, DEPUE RH AND JUDD HL. (1988). The early *in utero* oestrogen and testosterone environment of blacks and whites: potential effects on male offspring. *Br. J. Cancer*, **57,** 216–218.

HUTSON JM. (1985). A biphasic model for the hormonal control of testicular descent. *Lancet*, **2,** 419–421.

JOHN RADCLIFFE HOSPITAL CRYPTORCHIDISM STUDY GROUP. (1988). The clinical diagnosis of cryptorchidism. *Arch. Dis. Child.*, **63,** 587–591.

JOHN RADCLIFFE HOSPITAL CRYPTORCHIDISM STUDY GROUP. (1992). Cryptorchidism: a prospective study of 7500 consecutive male births, 1984-8. *Arch. Dis. Child.*, **67,** 892–899.

SCORER CG. (1956). The incidence of incomplete descent of the testicle at birth. *Arch. Dis. Child.*, **31,** 198–202.

UNITED KINGDOM TESTICULAR CANCER STUDY GROUP. (1994). Aetiology of testicular cancer: association with congenital abnormalities, age at puberty, infertility, and exercise. *Br. Med. J.*, **308,** 1393–1399.

WALKER AH, BERNSTEIN L, WARREN DW, WARNER NE, ZHENG X AND HENDERSON BE. (1990). The effect of *in utero* ethinyl oestradiol exposure on the risk of cryptorchid testis and testicular teratoma in mice. *Br. J. Cancer*, **62,** 599–602.